# Free-surface measurements in a cylindrical container using synthetic schlieren with extended paraxial range

**DOI:** 10.1007/s00348-026-04240-z

**Published:** 2026-08-20

**Authors:** Lyke E. van Dalen, Jan-Bert Flór, Jerry Westerweel

**Affiliations:** 1 Laboratory for Aero- & Hydrodynamics, Delft University of Technology, Mekelweg 2, 2628 CD Delft, The Netherlands; 2 Laboratoire des Écoulements Géophysiques et Industriels (LÉGI), Université Grenoble Alpes, CNRS, Grenoble INP, 3800 Grenoble, France

## Abstract

In this study, the *free-surface synthetic schlieren* (FS-SS) method, as proposed by Moisy et al. (*Exp. Fluids* **46** (2009) 1021), is extended to allow a wider application of the method. The reliance on small-angle paraxial approximation is removed, which allows for shorter distances of the camera to the free surface. Also, the presence of walls and non-constant depth of the liquid layer are accounted for. The difference between the original and modified approach is evaluated using a ray tracing approach. The extension of the method is demonstrated with measurements of the water surface in a cylinder for both rotating and non-rotating cases, i.e., surface waves due to impact, a surface depression due to a spin-up vortex, and the parabolic free surface in a rotating cylinder. The measured variations in surface elevation are as small as $\mathcal {O}$(10 μm) with a range in paraxial angle up to ±0.56 rad (${32.3}^\circ$).

**Supplementary Information:**

The online version contains supplementary material available at https://doi.org/10.1007/s00348-026-04240-z.

## Introduction

The measurement of a free liquid surface is relevant in many applications, and various approaches have been suggested, both intrusive and non-intrusive (Moisy et al. 2009; van Meerkerk et al. 2020; Gomit et al. 2022; Wang et al. 2024; Tamir et al. 2024). The *free-surface synthetic schlieren* (FS-SS) method, originally proposed by Moisy et al. (2009), relies on the refraction of light at the surface; it requires a patterned reference target on one side of the surface and a camera on the other side to record the image deformation of the observed pattern. Given the refractive index of the liquid and the distance between the undisturbed free surface and the reference target, the gradient ∇h of the deformed surface height can be determined. For this, the displacement field is measured between the undistorted and distorted reference images. The integration of ∇h then yields the actual free surface height *h*(*x*, *y*). This method provides accurate results, provided the approximations of (i) paraxial optical rays, (ii) small surface slopes, and (iii) small surface amplitudes are met (Moisy et al. 2009). Many other implementations of this method have been proposed to extend the range of applicability and also to lessen the restrictions of the original assumptions. Examples of this include: elimination of a required reference height (Li et al. 2021), the inclusion of patterns with a weak slope (Mermelstein and Dagan 2025), or the allowance of larger free-surface gradients (Kolaas et al. 2018).

The paraxial approximation has the advantage that a single conversion constant can be used to convert the image displacement $\delta \textbf{r}$ = (δx,δy) to surface gradient ∇h (Moisy et al. 2009), making this effectively a linear problem. This also means that, in practice, a relatively large working distance is needed for the recording camera. As demonstrated here, this is not always applicable, and relaxing the paraxial approximation implies that the conversion is no longer a constant and becomes a nonlinear problem.

In this paper, we present an extension to the approach of Moisy et al. (2009) and show the application of the method inside a liquid-filled cylindrical container for both rotating and non-rotating cases. For this setup, the assumption of (nearly) paraxial rays no longer holds due to the proximity of the camera to the free surface. Additionally, the reference target is not planar, but is located at both the bottom wall and the inner vertical wall of the cylinder. By providing a methodology for varying pattern-to-camera distances, the potential arises to use this technique for measuring near-wall waves (e.g., in the case of studying sloshing), or in the presence of submerged objects. Relaxation of the paraxial approximation allows for a wider use in laboratory settings where a large camera-to-pattern distance is simply not achievable.

The methodology of the technique is outlined in Sect. 2 and validated using a ray tracing approach. It is then applied to three illustrative experiments in Sect. 3. Firstly, a metal sphere is dropped to create small-amplitude surface waves; secondly, a tip vortex within a rotating cylinder is considered. The third case concerns a rotating cylinder with a parabolic water-air interface, where the largest gradients of the surface elevation occur at the sidewall (Greenspan 1968). This poses an additional challenge for near-wall measurements. The results and future applications are discussed in the conclusions.

## Method

**Fig. 1:**
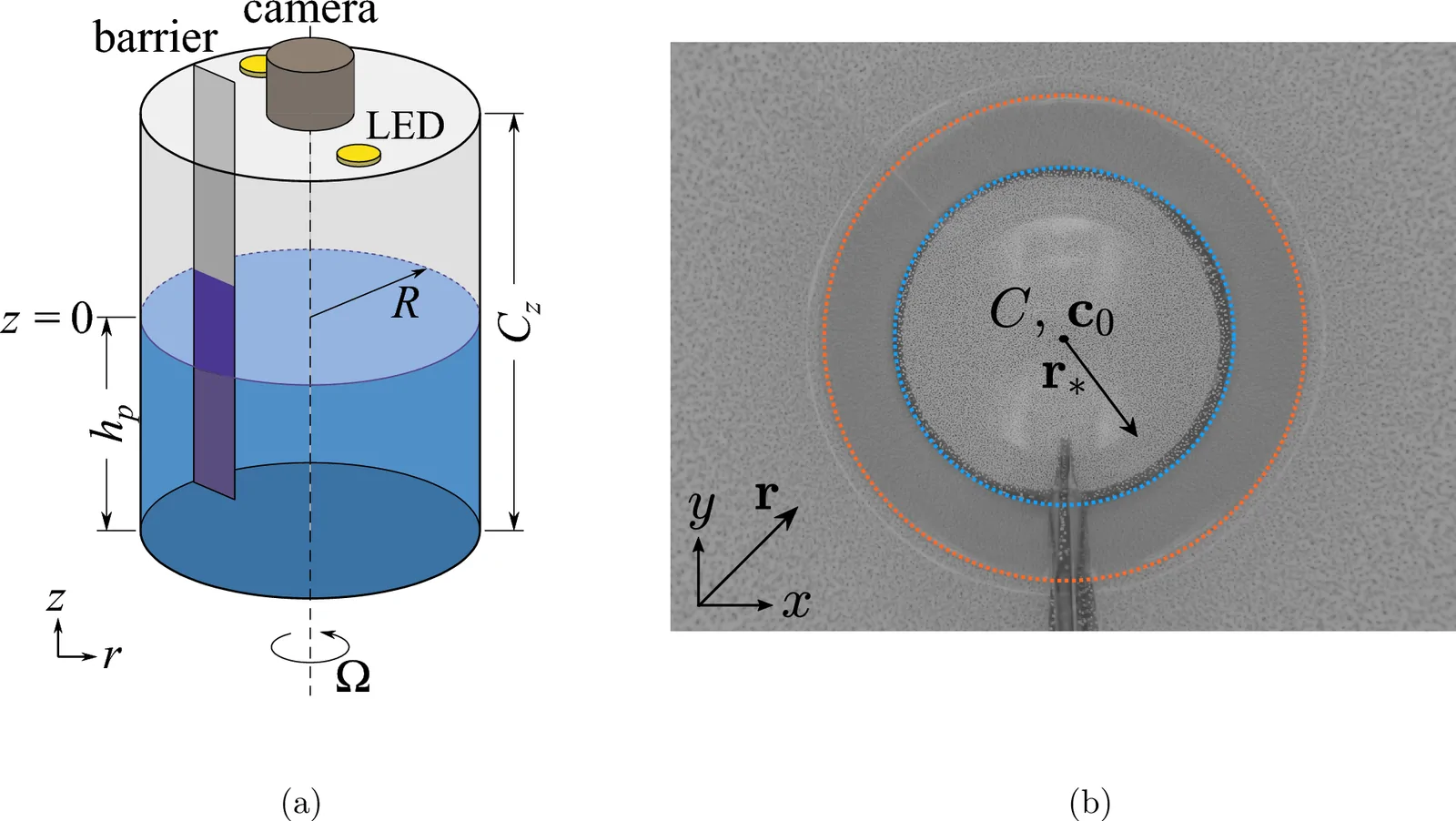
**a** Schematic of the rotating cylinder. (Symbols are explained in the text.) **b** Reference image of the dot pattern on the bottom (inside the dashed blue circle) and vertical cylinder wall (outside the dashed blue circle) through a flat water surface (i.e., non-rotating cylinder). The orange dashed circle marks the waterline at the vertical cylinder wall. The cylinder axis and optical axis are aligned, so that $\mathbf {c_0} = (C_x,C_y)$. (This is not a necessary requirement.)

The FS-SS method is applied in a water-filled cylinder mounted on a rotary table; see Fig. 1a. A camera is mounted on top of the open end of the cylinder, and co-rotates with the cylinder. The cylinder has an inner radius of *R* = 72 mm and a total height of 194 mm; the water depth is 80 mm, so that the camera is relatively close to the water surface. The bottom and vertical wall of the cylinder are covered with a laminated random dot pattern. Figure 1b shows an image of the dot pattern as viewed by the camera. In the original FS-SS method (Moisy et al. 2009), the gradient of the water surface ∇h is obtained from the observed displacement $\delta \textbf{r}$ of the dot images from:

1 $$\begin{aligned} \nabla h = -\frac{\delta \textbf{r}}{h^*}, \quad \text {with:}\quad \frac{1}{h^*} = \frac{1}{(1-n/n')h_p}-\frac{1}{C_z}, \end{aligned}$$

where *n* and $n^{\prime }$ are the refractive indices of air (*n* = 1.00) and water ($n^{\prime }$ = 1.33), respectively, and $$h_p$$ is the water depth. Our configuration complicates the application of the original FF-SS method. First, the camera is mounted relatively close to the water surface, while the water depth $$h_p$$ is not small with respect to the camera distance $$C_z$$; this means that the approximation of (nearly) paraxial viewing does not hold, and the conversion in (1) is no longer a linear problem. Due to the inclusion of the vertical wall, the maximum viewing angle with respect to the optical axis in our application increases to $$\beta _\textrm{max} (= R/(C_z-h_p))$$ = 0.63 rad (i.e., 36°), which can no longer be considered as small paraxial viewing. This is resolved by introducing a new parameter $$\delta \textbf{s}$$, which describes the offset of a point from its virtual object.

Secondly, for the bottom, we have a constant depth $h_p$ with respect to the undisturbed water surface, but this no longer holds for observed image displacement of dots on the vertical cylinder wall. For the vertical wall, we use the *effective depth* ${H}(x,y)$, which represents the actual interface-to-pattern distance for a non-flat geometry. This depth decreases when approaching the location where the water surface touches the cylinder wall (i.e., the waterline), where the effective water depth vanishes (${H}\rightarrow 0$) and the expression in (1) diverges, which is prone to large errors. A further complication that occurs when approaching the vertical wall is the presence of a meniscus on the water. This is further discussed in Sect. 3.2 and Appendix A.

Finally, the last complication lies in the fact that we use the cylinder in both rotating and non-rotating situations. When the cylinder rotates at a constant angular velocity Ω for a sufficiently long time, the water surface assumes a parabolic shape with a height (Greenspan 1968):

2 $$\begin{aligned} h_\textrm{par} = h_p+\frac{\Omega ^2}{2g}\left( {\textbf{r}_{*}}^2-\frac{1}{2}R^2\right) , \end{aligned}$$

where ${\textbf{r}_{*}}$ is the radial vector from the cylinder axis, and *g* the gravitational acceleration. Note that in (2) the total liquid volume in the cylinder is conserved. For an angular velocity Ω = 2.4 rad/s the difference in surface height at the center and at the wall is 1.5 mm (ignoring an additional rise of the surface due to a meniscus). We can determine the reference image of the dot pattern through a flat surface when the cylinder is not rotating and then compare it with the displacement of the dot images when the cylinder is rotating. We also consider transients by increasing the rotation rate of the cylinder from a constant (nonzero) rotation, thus spinning up the container fluid. In that case, it is more appropriate to use the reference image pattern while the cylinder is rotating, so that in the initial state the water surface is not flat.

**Fig. 2:**
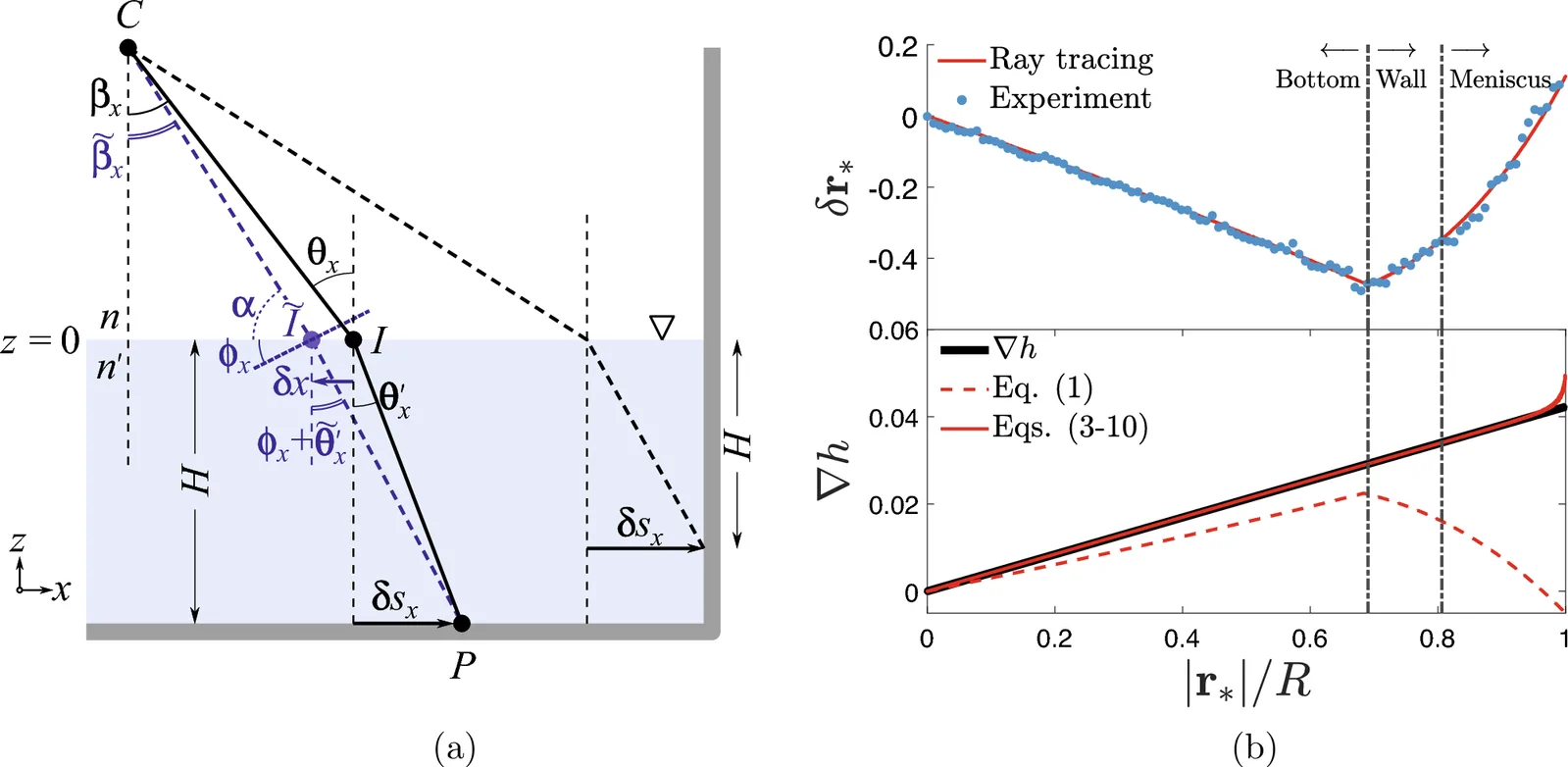
**a** Definition of ray tracing and surface gradient reconstruction parameters in the x-direction (not to scale); see text for explanation. **b** Dot pattern displacement $\mathbf {\delta r_*}$ in [mm] and the reconstructed surface gradient ∇h as a function of the normalized distance to the cylinder axis.

Considering these complications, we propose a method to solve for the surface height, while still only requiring geometrical parameters, camera location *C*, $$h_p$$, *R*, and the ratio of the refractive indices $n/n^{\prime }$. For this, consider the diagram of Fig. 2a. The undisturbed water surface defines the plane z=0 with a position $${\textbf{r}}=(x,y)$$ in that plane. The cylinder bottom is at $$z = -h_p$$. The cylinder axis is at $${\textbf{c}}_0 = (c_{0,x},c_{0,y})$$, and we define $${\textbf{r}_{*}}=\textbf{r}-\mathbf {c_0}$$ as the radial vector from the central axis of the cylinder.

Figure 1b shows the reference dot pattern on the bottom and vertical cylinder wall. The blue dashed circle represents the perimeter of the bottom, while the orange dotted circle represents the water-air interface at the vertical wall (i.e., waterline). The image of the bottom pattern covers 47% of the image area enclosed by the orange circle; this would imply a significant loss of information if only the pattern on the bottom (i.e., parallel to the undisturbed surface) is used. Please note that for the reconstruction of the water surface in the cylinder, it is not necessary for the optical axis to coincide with the cylinder axis; as a matter of fact, an origin for the displacement data can be chosen at any point in the image, even outside the area of interest.

For an observer in point *C* = $(C_x,C_y,C_z)$ each point *P* in the dot pattern is represented by its virtual object point *I* at the surface. In the case of a deformed water surface, a displacement $\delta \textbf{r}$ = $(\delta x,\delta y)$ is measured that is proportional to the gradient ${\nabla h}$ of the surface elevation *h*; see Fig. 2a.

We deal with the components of ∇h = $(\partial h/\partial x,\partial h/\partial y)^T$ separately, i.e., considering planar projections on the *yz*- and *xz*-planes, respectively. Below we present the equations for $\partial h/\partial x$, indicated by a suffix *x*, and illustrated in Fig. 2a; the expressions for $\partial h/\partial y$ are equivalent. The viewing angle, or paraxial angle, $\beta _x$ is given by

3 $$\begin{aligned} \beta _x = \arctan {\left( \frac{x-C_x}{{C_z-h_p}}\right) }, \end{aligned}$$

which is equal to the refracted angle $\theta _x$ with respect to the surface normal (here equal to the *z*-axis, or $[0\ 0\ 1]$). Using the Snell-Descartes law for refraction, we find the angle $\theta '_x$, i.e., $\sin \theta '_x = (n/n')\sin \theta _x$. This then gives the component $\delta s_x$ of the offset $\delta \textbf{s}= (\delta s_x,\delta s_y)$:

4 $$\begin{aligned} \delta s_x = H(x,y)\tan \theta _x'. \end{aligned}$$

The expression for ${H}(x,y)$ depends on the geometry of the container. To determine the effective depth, a ray is considered from camera *C* through *I* toward the pattern. Along this ray, *P* lies at the distance $|\delta \textbf{s}|$, where the ray intersects the pattern either at the bottom or at the wall. The appropriate expression for ${H}$ therefore depends on whether the ray reaches the bottom or intersects the cylindrical wall. This condition, as well as the adjusted ${H}$ in case of a wall intersection, is obtained by equating the location of *P* with that of the wall. From here, the conditions for ${H}$ and the adjusted distance of *P* toward the wall follow. In this case, a reference pattern on a cylindrical geometry where $(C_x,C_y)=\mathbf {c_0}$, the adjusted distance follows from solving the circle equation, $|P-{\textbf {c}}_0|^2=R^2$, as $R-|{\textbf{r}_{*}}|$ for $|P-{\textbf {c}}_0|>R$. Thus, ${H}$ is given by:

5 $$\begin{aligned} H(x,y) = \left\{ \begin{array}{cl} h_p & \text {for:}~|P-\mathbf {c_0}| \leqslant R \\ (R-|{\textbf{r}_{*}}|)\cot \theta ' & \text {for:}~|P-\mathbf {c_0}| > R \end{array} \right. \end{aligned}$$

with:

6 $$\begin{aligned} P= &  \textbf{r}+|\delta \textbf{s}|\frac{{\textbf{r}_{*}}}{|{\textbf{r}_{*}}|}, \quad \text {and:}\quad \nonumber \\ \theta '= &  \arcsin \left[ \frac{n}{n'}\sin \left\{ \arctan {\left( \frac{|{\textbf{r}_{*}}|}{C_z-h_p} \right) } \right\} \right] . \end{aligned}$$

The distance from the cylindrical center, $|P-\mathbf {c_0}|$, determines whether the dot is located on the vertical wall. The location of point *P* per $\textbf{r}$ is dependent on the refraction at $I = \textbf{r}$. Alternatively, $|P-\mathbf {c_0}|$ can be estimated from the measurement itself, as seen in Fig. 1b, where the location $|P-\mathbf {c_0}|=R$ is marked by the blue dashed circle.

When the point *P* lies on the vertical wall of the cylinder (for $|{\textbf{r}_{*}}|/R>$ 0.69 in Fig. 2b), the effective water depth ${H}$ decreases with increasing viewing angle β as it approaches the point where the water surface touches the cylinder wall.

For other geometries, only the expression in (5) must be adapted. As described above, the corresponding condition and adjusted distance are obtained by equating the expression for *P* in (6) with the wall location and solving for the distance to the wall. In the case of a general non-flat geometry or wall, ${H}(x,y)$ potentially needs to be specified for each section of the dot pattern geometry, possibly using ray tracing, provided the shape of the geometry is known.

The offset $\delta \textbf{s}$ is often neglected, as $|\delta \textbf{s}| \rightarrow 0$ for a camera positioned far from the surface and bottom ($C_z \gg h_p$) in the original paraxial approximation. However, ignoring the offset $\delta \textbf{s}$ can introduce significant errors, as it assumes ∇h is evaluated at *P* rather than *I*. Also, $\delta \textbf{s}$ is needed to evaluate (5) to obtain the proper value of the water depth ${H}(x,y)$.

Figure 2b shows the observed displacement of the reference pattern in Fig. 1b, when the cylinder rotates at a constant angular velocity (Ω = 2.4 rad/s), so that the liquid surface has a parabolic shape, given by (2), and |∇h| is directly proportional to the distance ${\textbf{r}_{*}}$ from the cylinder axis. The dot-image displacement $\mathbf {\delta r_*}$ in the bulk is as expected toward the cylinder axis (i.e., $\mathbf {\delta r_*}<0$) and directly proportional to the gradient of the water surface elevation. Figure 2b shows the reconstructed surface gradient $\partial h/\partial {\textbf{r}_{*}}$. The dashed red line represents the result obtained with the original expression (1). When we consider the bottom reference pattern, i.e., $|{\textbf{r}_{*}}|/R < 0.69$, the reconstructed surface gradient is indeed proportional to ${\textbf{r}_{*}}$, albeit underestimated; this is a direct consequence of ignoring the contribution of the offset $\delta \textbf{s}$. For $|{\textbf{r}_{*}}|/R > 0.69$ the reference points are located on the vertical cylinder wall, which implies that the *effective* water depth ${H}$ *decreases* when the measurement location approaches the waterline (orange dashed curve in Fig. 1b), leading to erroneous results when using (1). The reconstruction of ∇h using the following proposed method in red yields the proper slope up to very close to the wall.

When the surface deforms, the virtual object point *I* at the undisturbed surface (z=0) of a point *P* on the reference pattern is displaced to a virtual object position $\tilde{I} = I+\delta \textbf{r}$, with: $\delta \textbf{r}= (\delta x,\delta y)$; see Fig. 2a. Below we explain the adapted reconstruction for the displacement component δx and $\partial h/\partial x$, while the reconstruction for $\partial h/\partial y$ is similar.

The angle $\tilde{\beta }_x$ follows from:

7 $$\begin{aligned} \tilde{\beta _x} = \arctan {\left( \frac{x-C_x+\delta x}{{C_z-h_p}}\right) }, \end{aligned}$$

where $\tilde{\beta _x} \approx \beta _x$ for small displacements δx. Given the depth ${H}$, displacement δx, and offset $\delta s_x$ we find:

8 $$\begin{aligned} &  \frac{\delta s_x-\delta x}{H} = \tan (\phi _x+\tilde{\theta _x'}) \quad \Rightarrow \quad \nonumber \\  &  \quad \phi _x + \tilde{\theta '_x} = \arctan \left( \frac{\delta s_x - \delta x}{H}\right) , \end{aligned}$$

where $\tilde{\theta }_x'$ is the angle between the ray from *P* to $\tilde{I}$ and the normal of the deformed surface, and $\phi _x = \arctan (\partial h/\partial x)$ the local angle of the surface at $\tilde{I}$; see Fig. 2a The angle $\tilde{\theta _x'}$ relates to the angle $\tilde{\theta }_x$ of the refracted ray between $\tilde{I}$ and *C* via the Snell-Descartes law, i.e., $n'\sin \tilde{\theta }_x' = n\sin \tilde{\theta }_x$. Given that: $\phi _x+\alpha +\tilde{\theta }= \pi /2$, and: $\alpha = \pi /2 - \tilde{\beta }$, we arrive at:

9 $$\begin{aligned} \phi _x = \arctan \left( \frac{\delta s_x-\delta x}{{H}}\right) -\arcsin \left[ \frac{n\ }{n'}\sin \left( \tilde{\beta }_x-\phi _x\right) \right] . \end{aligned}$$

This expression is implicit in $\phi _x$, and can be solved iteratively, although generally $|\tilde{\beta }_x| \gg |\phi _x|$. The surface gradient $\partial h/\partial x$ then follows from $\tan \phi _x$. Similarly, $\partial h/\partial y = \tan \phi _y$ is found. Given both $\partial h/\partial x$ and $\partial h/\partial y$, the deformed surface $h_{\textbf{r}+\delta \textbf{r}}$ is found by integration, using volume conservation for the integration constant. It is noted that the approach for $\partial h/\partial x$ is not fully independent of $\partial h/\partial y$, and *vice versa*; however, for small variations of the surface elevation, the expression in (9) is to leading order correct. In the present setup we find that the projected surface slopes typically deviate less than about 3.5 mrad (0.2°) for an actual surface slope angle of 0.18 rad (10°).

In the approach described above, it is implicitly assumed that the change in height of the water level, $\Delta h = h-h_p$, is small with respect to the water depth. However, as we approach the waterline, the effective depth ${H}$ decreases closer to the cylinder wall, causing the relative change Δh/H to increase. Ignoring the contribution of Δh to the effective water depth ${H}$ is of minor importance when considering the *relative* change in water height (such as in Case 2), since Δh is small. However, it must be taken into consideration when determining a significant increase in the absolute water height near the wall. This would be the case for measuring the absolute water height in the rotating cylinder where the water surface assumes a parabolic shape (2). Here, the maximum gradient ∇h and surface height *h* occur at the vertical cylinder wall, where also the paraxial viewing angle β is large (see Case 3). A solution to this is to incorporate an *a priori* estimate for the depth ${H}$ in the deformed situation and the corresponding correction for the displacement $\delta \textbf{r}= (\delta x,\delta y)$, i.e.,

10 $$\begin{aligned} \widetilde{H} = H+\Delta h|_{{\textbf {r}}+\delta \textbf{r}}, \quad \text {and:}\quad \widetilde{\delta x} = \delta x-\tan (\tilde{\beta }_x) \Delta h|_{{\textbf {r}}+\delta \textbf{r}}, \end{aligned}$$

and similarly for δy. This can be used in Eqs. (5–9) for ${H}$ and δx, respectively. Equation 10 is only applied in Case 3.

### Ray tracing

To demonstrate the differences of the modified method with respect to the original method and to identify where possible errors may occur, we apply ray tracing to generate displacements $\delta \textbf{r}$ for model surfaces representative of the three cases mentioned before. We consider an optical pin-hole model with the optical rays in the vertical *yz*-plane only that cross either a planar or a deformed water surface. This yields numerical results for $\delta \textbf{r}$ in the case of ideal measurements, as for example shown in Fig. 2b, which are then introduced into the original FS-SS reconstruction in (1) as well as the proposed modified method. This allows us to understand the errors induced by each of the two methods when the assumptions regarding rays with small paraxial angles and small variation in surface deformation with respect to water depth are no longer valid.

For the evaluation of Case 1, a deformed water surface with the shape of a sinusoidal wave around z=0 is considered, i.e., $h_s= h_p+A\sin (2\pi r/\lambda )$, where *A* and λ are the amplitude and wavelength, respectively, that mimic the measured waves at the surface shown in Fig. 4, with *A* = 0.10 mm and λ = 10 mm. For Case 2, we consider a Gaussian profile to represent the surface depression associated with the vortex, i.e., $h_g = h_p+a\exp [-\frac{1}{2}(r-\mu )^2/\sigma ^2]+v_0$, where the offset $v_0$ accounts for volume conservation. The values of the parameters that correspond to the result in Fig. 5a are: a=-1.2 mm, μ = 48 mm, σ = 10 mm, and $v_0 = 0.207$ mm. For Case 3, we consider the parabolic surface in (2) with: Ω = 2.4 rad/s, and *g* = 9.81 $\hbox {m/s}^2$.

**Fig. 3:**
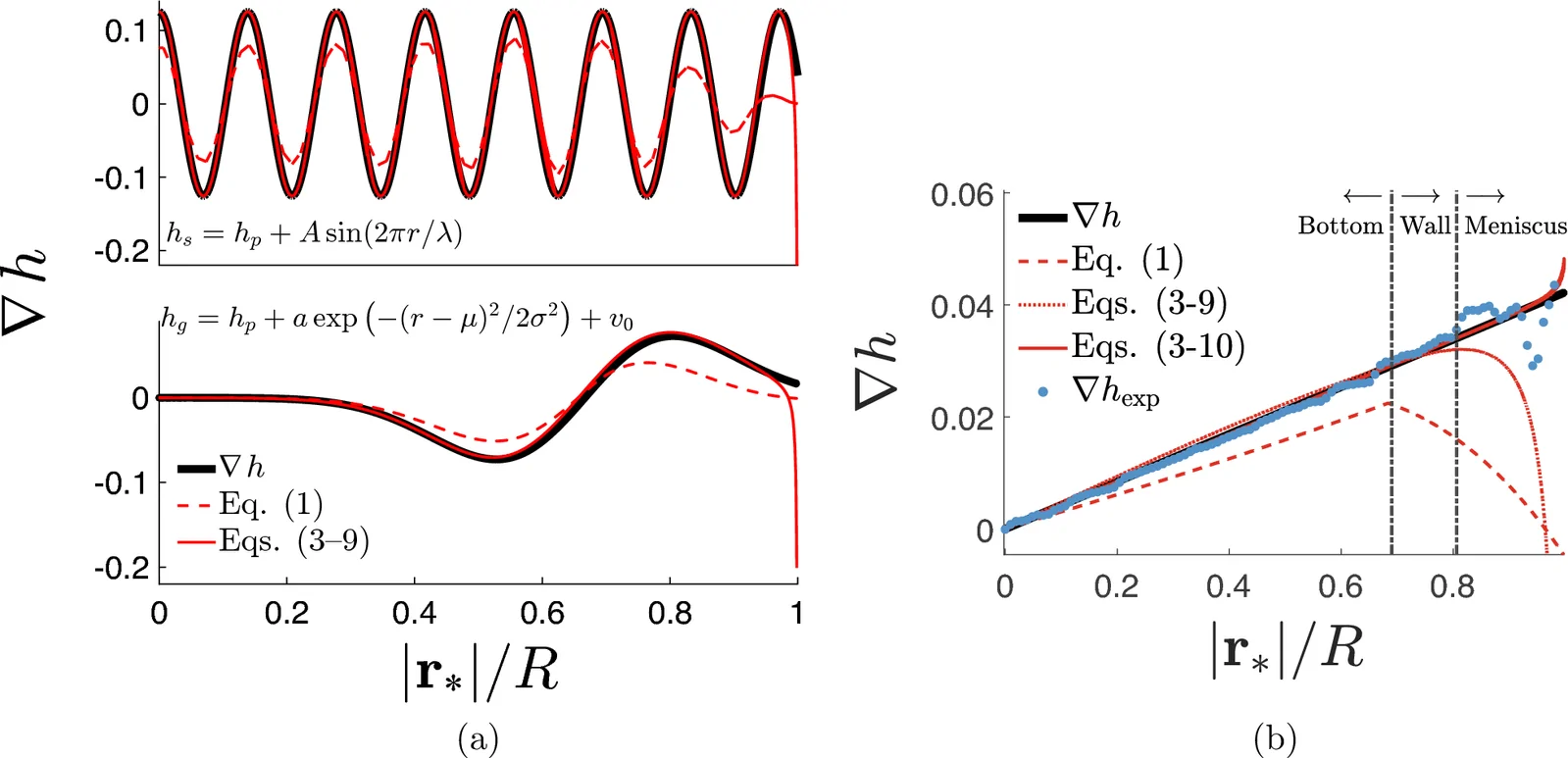
Comparison between the gradient ∇h of the deformed interface (solid black lines) and its reconstruction, following either (1), represented by red dashed lines or Eqs. (3–9/10) in solid red lines. **a** Sinusoidal surface deformation $h_s$ (top graph) and a Gaussian surface depression $h_g$ (bottom), representing Cases 1 & 2 (see text), and **b** parabolic deformation $h_\textrm{par}$, representing Case 3 (see text). The result from the slice in Fig. 6a is included; see text for details.

Figure 3a shows the ideal results for Cases 1 & 2. Note that the original reconstruction using (1), where paraxial imaging is assumed, for the sinusoidal surface deformation appears to return the proper wavelength, except for close to the vertical cylinder wall. However, the amplitude is significantly underestimated over the full range. The same is observed for the Gaussian depression where the original reconstruction appears to yield approximately the proper shape, but with a severe underestimation of the magnitude of the surface gradient; this yields a significant underestimation of the actual depth of the surface depression. For both Cases 1 & 2, the proposed method (3–9) performs considerably better. The reconstructed gradient remains within 5% of |∇h| up to $|{\textbf{r}_{*}}|/R=0.98$ and $|{\textbf{r}_{*}}|/R=0.95$ for $h_s$ and $h_g$, respectively.

For Case 3, the results are presented in Fig. 3b. As shown, even for the parabolic surface, we are able to reconstruct within 5% of |∇h| up to $|{\textbf{r}_{*}}|/R=0.93$. The importance of the correction in (10) is highlighted, which shows a clear improvement using the ray tracing approach compared to using (3–9) without the correction.

## Application

The method and approach explained in Sect. 2 is applied to three cases with a similar geometry, i.e., a cylindrical container filled with water. In the first case, a metal ball is dropped into the stationary (non-rotating) cylindrical container. In the second case, the cylinder is rotating at a constant angular velocity $\Omega _1$, so that the image of the reference target is taken through a parabolic surface. When the rotational velocity of the cylinder is increased to $\Omega _2$, a vortex forms at the tip of a vertical barrier at the inner vertical wall of the cylinder. The lower pressure in the core of the columnar vortex creates a depression at the free surface. In the third case, we consider the parabolic surface in a rotating cylinder without barrier. The reference image for the dot pattern is then that of a flat surface for a non-rotating cylinder.

The cylinder is made of PMMA and has an inner radius of *R* = 72 mm. The height of the water level is *h* = 80 mm. In Cases 1 & 2 there is a barrier mounted on the inner wall of the cylinder over the full height of the cylinder. The barrier extends 25 mm in the radial direction of the cylinder and has a thickness of 2 mm. In Case 2, where the cylinder spins up, the barrier generates the vortex. (It is noted that the barrier has no specific function for the test in Case 1.) A digital camera (GoPro Hero 7) with a 2704×2028 pixel (px) image format and a recording frame rate of 30 Hz is mounted above the center of the cylinder (thus, $\mathbf {c_0}= (C_x,C_y)$) at height $C_z$ = 194 mm above the cylinder bottom. The largest paraxial angle is β = 0.56 rad (32.3°). Illumination is provided by two LED’s (Lume Cube 2.0) positioned either at the top in Case 2 or the sides in Cases 1 and 3. The cylinder, cameras, and LED’s are mounted on a rotary stage (Zaber X-RSB120AD-E01). The influence of measurement uncertainties in the geometry is estimated using ray tracing, as the effect also depends on the displacement field and therefore varies for each Case. The generated $\delta \textbf{r}$ values together with the geometrical parameters ± accuracy bounds are used in the reconstruction of ∇h. Conservative uncertainties of 1, 0.5, and 0.1 mm are assumed for $h_p$, $C_z$, and *R*, respectively. In the bulk region, the maximum error in |∇h| among the three cases is $1.7\times 10^{-4}$ for $h_p$, $5.7\times 10^{-5}$ for $C_z$, and $5.8\times 10^{-5}$ for *R*. In the sidewall region, the corresponding values are $2.2\times 10^{-4}$ for $h_p$, $1.1\times 10^{-4}$ for $C_z$, and $4.2\times 10^{-4}$ in *R*.

Laminated paper sheets with printed ‘random’ speckle patterns are attached to the bottom and vertical walls of the cylinder. The dots in the speckle pattern have a typical diameter of 0.4 mm, with a 50% coverage. On the bottom of the cylinder, they appear with a diameter of approximately 3 pixel units (px) on the camera sensor. The equivalent size of a pixel projected on the planar water surface is 89 μm. Assuming an accuracy of 0.1 pixel for the displacement, this converts differently toward ∇h depending on $\textbf{r}$: in the bulk, it translates to an average error in |∇h| of 0.6$\times 10^{-3}$, and 1.3$\times 10^{-3}$ for the sidewall ($0.69<|{\textbf{r}_{*}}|<0.98$).

The raw images are pre-processed using a 3×3 px min-max filter (Adrian and Westerweel 2011), which removes reflections on the water surface. After this, the displacement of the dot pattern is evaluated using a spatial cross-correlation between the reference image and the distorted image using a multigrid PIV algorithm.

The first pass uses 64×64 px interrogation windows, followed by 32×32 px interrogations, both on a Cartesian grid with a 50% overlap between adjacent interrogations. To decrease the vector spacing, a final 32×32 px pass with 75% overlap is done. The resulting image displacements are converted to physical displacements: $\delta \textbf{r}= (\delta x, \delta y)$.

Since the recording of the reference image may be sensitive to external influences (Moisy et al. 2009), correlation averaging is used to reduce fluctuations in the initial conditions. Multiple frames from a recording of the initial state are correlated with individual frames of the transient measurements, or multiple frames of the steadystate measurements. All data are post-processed using a 3×3-px median test to identify and remove spurious displacements (Westerweel and Scarano 2005). For the steadystate measurements, the maximum absolute dot-image displacement is around 4 px, with a percentage of detected spurious displacements of approximately 0.7%; the spurious data are replaced by interpolation from the valid surrounding displacement data.

The displacements are converted to the surface elevation gradient ∇h using the method described in Sect. 2, and the Matlab function intgrad^1^ is applied to integrate ∇h to yield the surface elevation *h*. The integration constant for the height *h* is obtained using volume conservation of the total amount of liquid in the container.

### Case 1: dropping a metal ball

**Fig. 4:**
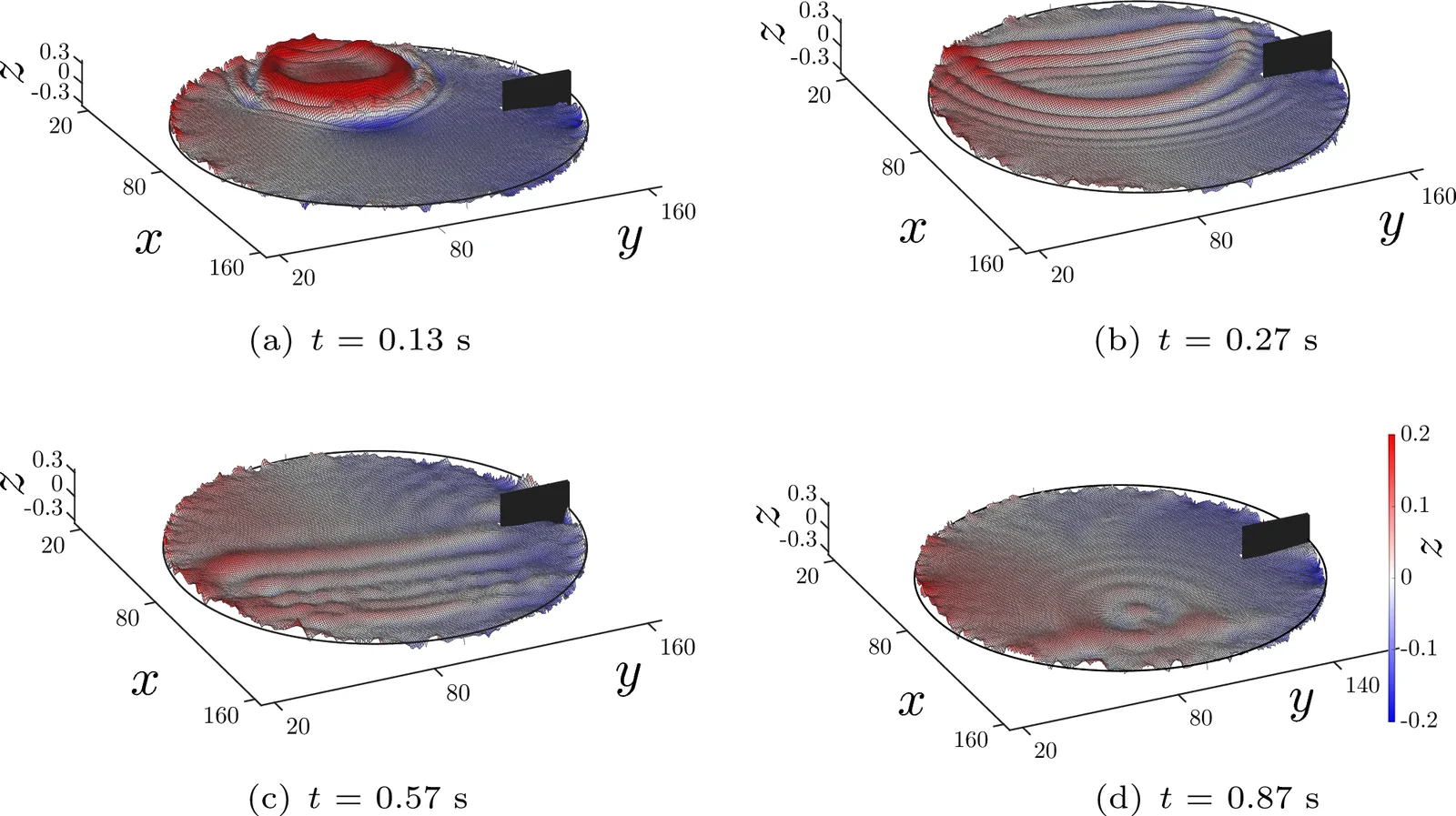
The measured water surface height in [mm] within the cylinder at *t* = 0.13, 0.27, 0.57, and 0.87 s after impact of the sphere with the water surface. Waves travel from the impact location. After the waves reflect from the opposite side of the cylinder, interference patterns can be observed. Note also the waves reflected by the barrier (black) at *t* = 0.57 s. All dimensions are in [mm]; note that the scale of the *z*-axis is enlarged for visibility. Contour plots in the *xy*-plane and a corresponding movie (movie 1) are available as supplementary material.

A metal ball with a 5 mm diameter is dropped from a height $C_z$ above the cylinder bottom in still water. The transient of the water surface after impact is measured. From the recorded 10-second video, 50 video frames of the reference pattern through quiescent water (thus before impact) are correlated with each individual video frame of the transient after impact using correlation averaging. The results for times *t* = 0.13, 0.27, 0.57, and 0.87 s after impact are plotted in Fig. 4; a full movie of the transient is available as supplementary material (movie 1). The metal ball impacts the surface at $|{\textbf{r}_{*}}|/R=0.63$, and the reconstructed surface height shows the characteristic shape of the impact waves. These waves have an amplitude of A≈ 0.1 mm and wavelength $\lambda \approx$ 1 cm. Although the (black) barrier visible in Fig. 4 has no special function in this case, one can observe waves reflected from its surface (Fig. 4, *t* = 0.57 s). A quantification of the waves in Fig. 4b is provided in the supplementary materials, along with contour plots for all panels in Fig. 4.

### Cases 2 & 3: rotating cylinder

**Fig. 5:**
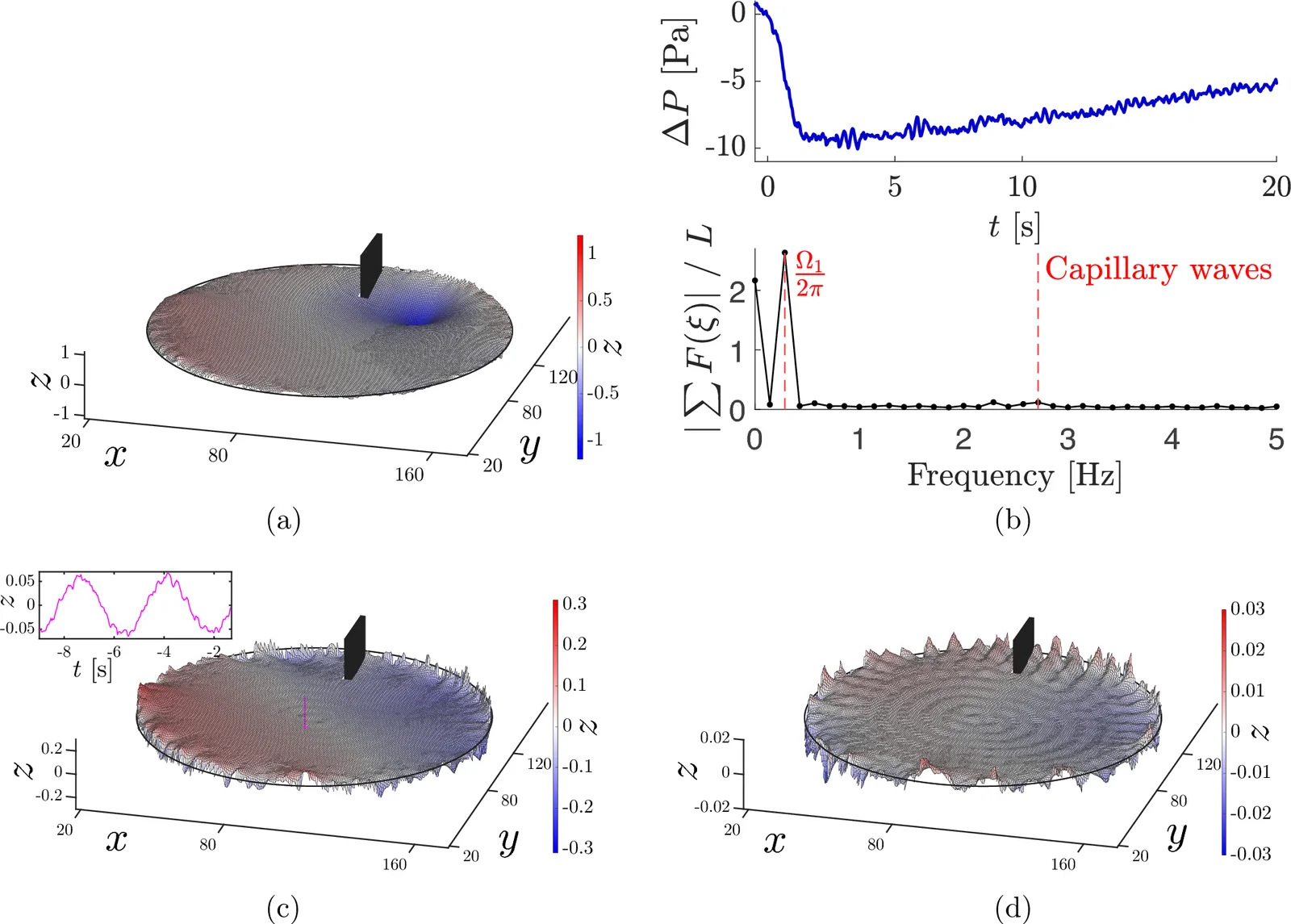
Measurement of the relative change in surface height after spin-up of a rotating cylinder with a barrier. All dimensions in **a**, **c** & **d** are in [mm]; the scale of the *z*-axis is enlarged for visibility. **a** Snapshot of the depression of the water surface due to the lower pressure in the vortex at *t* = 2.3 s. A corresponding movie is available in the supplementary material (movie 2), along with a contour plot in the *xy*-plane, and a height profile of the vortex. **b** The pressure drop ΔP in the vortex core as a function of time (top). Note the small-amplitude oscillations (with an equivalent amplitude of variations in surface height $\mathcal {O}$(10 μm)) with a frequency of 2.5–3 Hz. These fluctuations appear to be capillary waves, shown in **d**. (bottom) The sum of Fourier signals of each point in time for t<0, normalized by the duration *L* of 2 full rotations shows a distinctive peak at a frequency *f* (= $\Omega _1/2\pi$) = 0.29 Hz and a smaller peak at *f* = 2.71 Hz. The latter corresponds to the frequency of the oscillations shown in **d**. **c** A wave traveling around the tank due to a slight tilt of the rotation axis with *f* (= $\Omega _1/2\pi$) = 0.29 Hz. The inset shows the wave amplitude as a function of time at $|\textbf{r}_*|/R$ = 0.25. **d** Capillary waves with amplitudes of $\mathcal {O}$(10 μm) measured at *f* = 2.71 Hz.

During rotation, the method is first applied to measure the pressure in the core of a vortex formed at the tip of a barrier during the spin-up of the cylinder as it rotates. We increase the angular velocity of the cylinder at *t* =0 s from a steadystate with $\Omega _1$ = 1.8 rad/s to $\Omega _2$ = 2.4 rad/s with an angular acceleration of $\dot{\Omega }$ = 0.75 $\hbox {rad/s}^2$. The relative shift of the dot images between $\Omega _1$ and $\Omega _2$ is measured as $\delta \textbf{r}$, thus referring to the relative change in height. In the initial state, 105 frames (covering a single full cylinder rotation) are used for the correlation averaging. As the rotational velocity increases, a vortex starts to grow at the tip of the barrier, as shown in Fig. 5a as well as movie 2 of the supplementary material. Using hydrostatic equilibrium, $\Delta P = \rho g\Delta h$, the pressure difference in the core of the vortex can be obtained, assuming a density ρ = 998 $\hbox {kg/m}^3$, and gravitational acceleration *g* = 9.81 $\hbox {m/s}^2$; see Fig. 5b. Small amplitude oscillations in the pressure difference are noticeable in the pressure signal; these are physical oscillations in the surface height. This is characterized by computing the Fourier transform F(ξ) at each point in time over 2 rotational periods. The absolute amplitudes |F(ξ)| are summed over all frames and normalized by the duration *L* of 2 full rotations of the included samples. The result is shown in Fig. 5 and shows one dominant peak at a frequency *f* (=$\xi /2\pi$) = $\Omega _1/2\pi$ (= 0.29 Hz), and two smaller peaks at 2.29 and 2.71 Hz.

The specific mode for the dominant peak at *f* = 0.29 Hz is isolated and shown in Fig. 5c. This appears to be a slight sloshing motion of the water surface, visible by a higher level (red) to the left and a lower level (blue) to the right of the cylinder axis. The inset shows the height of the water surface for this motion as a function of time at $|{\textbf{r}_{*}}|/R=0.25$. The typical amplitude is $\mathcal {O}$(60 μm). It appears that this slight sloshing can be explained by a very small misalignment of the vertical cylinder axis of 3.3 mrad (0.19°) with the vertical.

The isolated mode at a frequency of *f* = 2.71 Hz is shown in Fig. 5d. The wave pattern appears as capillary waves, likely due to vibration from the rotating table. The waves have an amplitude of $\mathcal {O}$(10 μm). Using ray tracing, the largest corresponding displacement $|\delta \textbf{r}|$ would be $\mathcal {O}$(110 μm), equivalent to 1.2 px for this type of wave (A=10 μm and λ=10 mm). Thus, we consider these to be the smallest waves that can be measured in the present setup. The Fourier transform during acceleration (not shown) indicates waves with a wider range of frequencies, introduced by increasing the angular velocity Ω of the rotary table. The signal at the rotational frequency Ω is filtered out in order to remove the running sloshing wave in Case 2. The smaller amplitude contributions are kept and cause the small oscillations in the pressure signal (Fig. 5b).

**Fig. 6:**
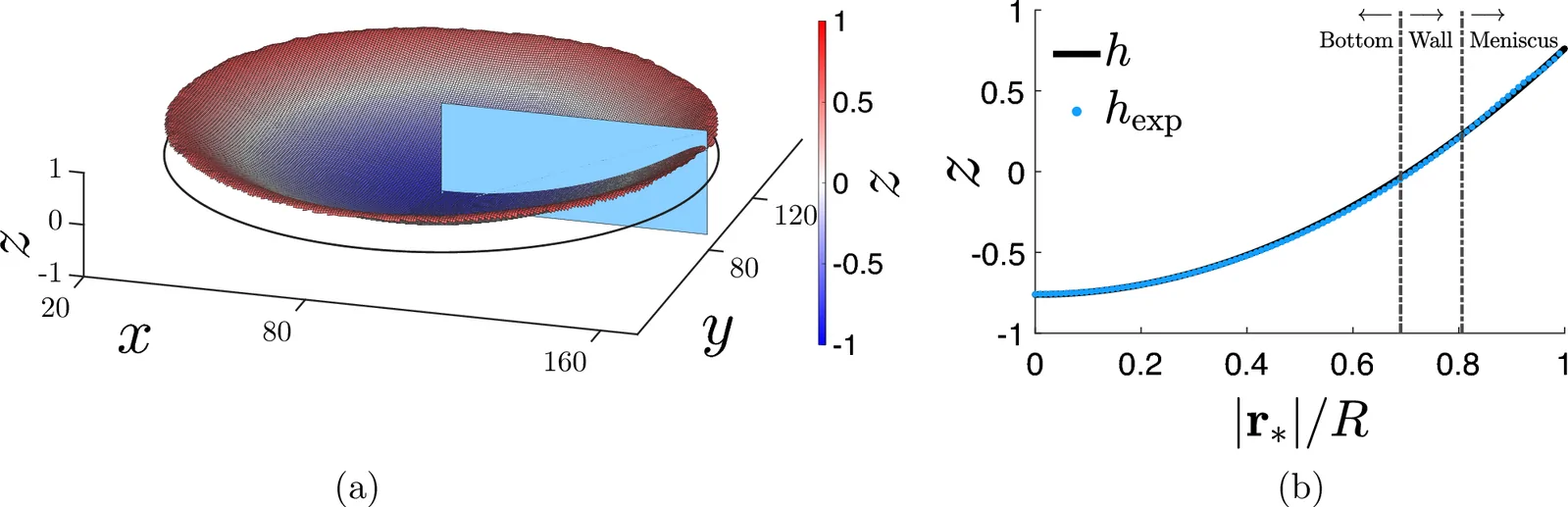
The reconstructed surface height *h*(*x*, *y*) for the rotating cylinder (Ω = 2.4 rad/s) without barrier (Case 3; see text). The height of surface in the blue slice along the *x*-axis in **a** is shown in **b** as $h_\textrm{exp}$ (blue dots), together with the expected height (black solid line) in (2). The measured image displacement $\delta \textbf{r}$ and surface gradient ∇h for the slice are shown in Fig. 2b and Fig. 3b, respectively. All dimensions are in [mm]; the scale of the *z*-axis is enlarged for visibility.

Finally, for Case 3 we measure the steadystate surface height *h* of a cylinder rotating at an angular velocity of Ω = 2.4 rad/s relative to a non-rotating undisturbed surface. As mentioned before, this is rather challenging due to the higher surface gradient, as well as the larger surface elevation difference and large paraxial angle at the vertical wall. For this measurement, we perform correlation averaging over 105 frames at Ω = 0 rad/s and 105 frames at Ω = 2.4 rad/s. The measured surface elevation is shown in Fig. 6a, while in Fig. 6b the surface profile, taken in a slice through the cylinder axis in the direction of the *x*-axis, is compared with the theoretical profile (2). The maximum difference between the measured and theoretical results is only 17 μm at $|{\textbf{r}_{*}}|/R = 0.92$. This corresponds to 3% of local value of $h_\textrm{par}$.

The measured surface elevation gradient ∇h for this same slice is plotted in Fig. 3b. From this, it becomes clear that the measured gradient exhibits increasing fluctuations for $|{\textbf{r}_{*}}|/R>0.8$ originating from deviation in $\delta \textbf{r}$. Close to the waterline at the vertical wall, small variations in the measured $\delta \textbf{r}$ lead to larger deviations in the measured gradient than in the bulk of the cylinder. In addition, the meniscus that exists at the waterline causes a further increase in the surface gradient and height, which in turn alters the displacement $\delta \textbf{r}$. This is also noticeable in Fig. 2b for $|{\textbf{r}_{*}}|/R>0.81$ (which is the extent of the meniscus; see Appendix A) where $|\delta \textbf{r}_\textrm{exp}|$ exceeds the expected displacement $|\delta \textbf{r}|$ in the absence of a meniscus.

We note that the actual height of waterline may also be affected by pinning of the contact line (De Gennes 1985) as the water level at the vertical cylinder wall rises from the non-rotating to the rotating state. This is actually dictated by local wall surface structure. We found this in some of the measurements, where the waterline remained pinned or did not rise to the expected level. Further details are given in Appendix A.

## Conclusions

In the present study, the free-surface synthetic schlieren (FS-SS) technique, originally proposed by Moisy et al. (2009), is extended to account for non-constant depth and to remove the need for a paraxial approximation. This modification allows for a wider use of the technique, relaxing the difficult-to-achieve camera-to-pattern distance required for classical FS-SS, and introduces the possibility for more complex geometries, while still using few geometrical parameters.

We use a ray tracing approach to elucidate the difference between the original approach and the present approach; in particular this shows that measurements can be taken when the reference pattern is not parallel to the undisturbed surface and where the imaging covers a substantial range in paraxial angles. The approach makes it possible to use a non-constant water depth, and it can resolve the gradient of the water surface elevation close to the waterline, where the water depth vanishes.

The approach is demonstrated by measuring the water surface inside a cylinder geometry, performing three distinct experiments with paraxial angles up to 0.56 rad (32.3°), namely: (i) waves generated by impact, (ii) a vortex-induced surface depression, and (iii) a parabolic surface, with the latter two inside a rotating cylinder.

The measurements in Cases 1 & 2 resolve capillary waves and a free-surface depression down to waves of $\mathcal {O}$(10 μm), while the results of Case 3 extend over a range of more than 1.5 mm, including steep surface gradients near the waterline at the vertical wall of the cylinder.

Overall, the results show strong potential for capturing the free surface shape in experiments where the camera is located rather close to the reference pattern and/or in the vicinity of walls that protrude the liquid surface. This opens the door to applying the FS-SS technique to sloped surfaces, submerged objects, sloshing flows, and other experimental configurations where a large camera-to-pattern distance cannot be achieved.

## Supplementary Information

Below is the link to the electronic supplementary material.Supplementary file 1 (mp4 7850 KB)Supplementary file 2 (mp4 64035 KB)Supplementary file 3 (pdf 4221 KB)

## Figures and Tables

**Fig. 7:**
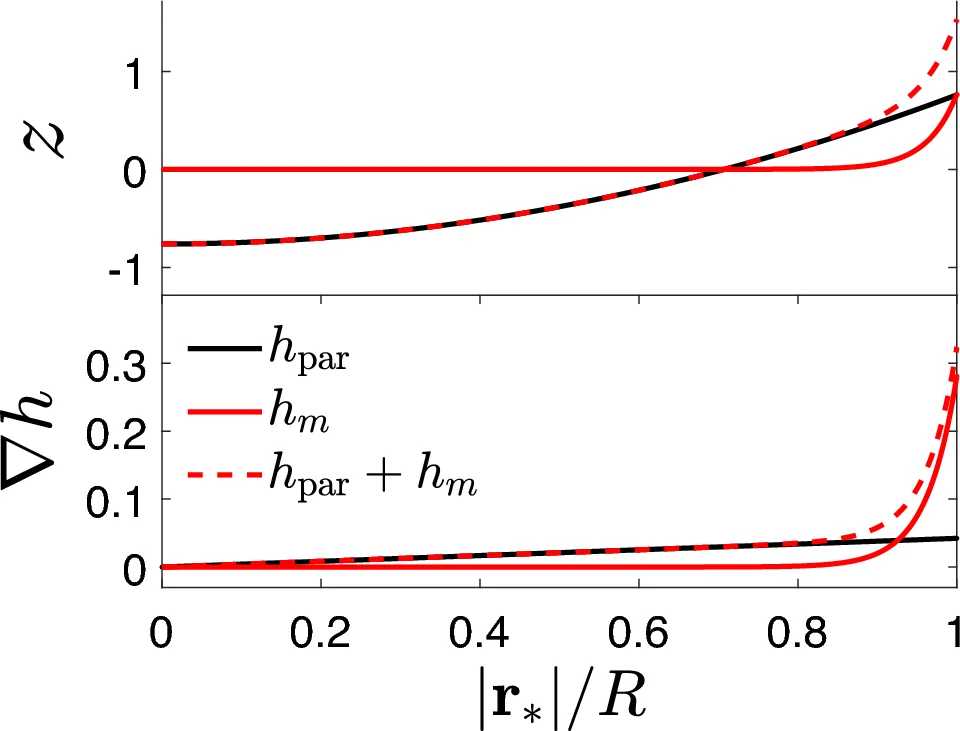
(top) The expected height of the parabolic surface $h_\textrm{par}$ in the rotating cylinder for Ω = 2.4 rad/s according to (2) and of the meniscus $h_{m}$ in (11), both with respect to the surface height $h_p$ in the non-rotating cylinder, as a function of the distance $|{\textbf{r}_{*}}|$ from the cylinder axis relative to the cylinder radius *R*. (bottom) The corresponding surface gradients ∇h of the parabolic surface $h_\textrm{par}$, the meniscus $h_m$, and the combined $h_m+h_\textrm{par}$

## Data Availability

No datasets were generated or analyzed during the current study.

## Meniscus

The height of the meniscus in the cylinder relative to the undisturbed (plane) water surface is given by Brennen (2006):

11 $$\begin{aligned} h_m = h_S \cot \varepsilon \exp \left( -\frac{R-|{\textbf{r}_{*}}|}{h_S}\right) \quad \text {with:}\quad h_S = \left( \frac{S}{\rho g}\right) ^{1/2}, \end{aligned}$$

where $R-|{\textbf{r}_{*}}|$ is the distance from the cylinder wall, *S* the surface tension, ρ the fluid density (998 $\hbox {kg/m}^3$ for water at room temperature), *g* = 9.81 $\hbox {m/s}^2$ the gravitational acceleration, and ε the water contact angle with the solid/gas interface at the wall.

The water surface tension and contact angle of PMMA with water and air are 72.80 mN/m and 1.296 rad (74.25°) at 293 K, respectively (Zdziennicka et al. 2017). For the present measurements, this yields $h_S$ = 2.7 mm and a maximum height of the meniscus of $h_S\cot \varepsilon$ = 0.76 mm. This value is of the same order of magnitude as the surface height variations $h_\textrm{par}$ measured in Case 3. The effect of the meniscus becomes apparent by summing the two: $h_\textrm{par}+h_m$. Note that this does not represent the exact surface height, as the two effects are coupled, such as the effect of the existing parabola on the contact angle, which is neglected here. However, we conjecture that, specifically for the gradient (since $|\nabla h_\textrm{par}|\ll |\nabla h_m|$ near the wall), this is a reasonable assumption.

The additional surface height due to the meniscus, see Fig. 7, causes a significant change in the perceived depth close to the wall, both in the undeformed and deformed situation. Moreover, because the meniscus develops over a much shorter length scale, it reaches a gradient that is over 6 times higher than $\nabla h_\textrm{par}$. We expect the effect of the meniscus to become significant at $|{\textbf{r}_{*}}|/R>0.81$, where its gradient exceeds 5% of $|\nabla h_\textrm{par}|$. For larger experimental setups, the effects are likely to be negligible.

## Footnotes

1 John D’Errico (2025). Inverse (integrated) gradient (https://www.mathworks.com/matlabcentral/fileexchange/9734-inverse-integrated-gradient), MATLAB Central File Exchange. Retrieved October 5, 2025.
