# Supplementary material for: Free-surface measurements in a cylindrical container using synthetic schlieren with extended paraxial range
Source: Exp Fluids. 2026 Aug 20;67(9):137. doi: 10.1007/s00348-026-04240-z (PMC13493441; doi:10.1007/s00348-026-04240-z)
Supplement: Supplementary file 1 [file 348_2026_4240_MOESM3_ESM.pdf]

Supplementary material: Free-surface measurements in a cylindrical container using synthetic schlieren with extended paraxial range

Lyke E. van Dalen, Jan-Bert Flór, Jerry Westerweel

Two dimensional view

Within the main manuscript, the focus is on the three-dimensional representation of the surface height measurements. Here, we present a complementary view based on slices and contour plots on the height data from Figs. 4 and 5 of the main manuscript.

Figures 1 and 2 correspond to Case 1 from Fig. 4 of the main manuscript. The height profile is extracted along a slice $y = -0.136x + 102$ [mm], which passes through the impact location and propogating wavefront. This profile is used to quantify the waves shown in Fig. 1(b), where the first and second wavepackets are measured to have a wavelength λ of 10.2 and 9.1 mm, respectively. The maximum amplitude is measured to be 0.055 mm. Figure 2 shows contour plots of the surface height in the xy-plane, corresponding to Fig. 4 of the main manuscript.

Figure 3 elaborates on Fig. 5 from the main manuscript. It presents a contour plot of the surface height together with the profile along the slice $y = 1.864x + 74$ [mm], which passes through the cylindrical axis $\mathbf{c}_0$ and the center of the vortex.

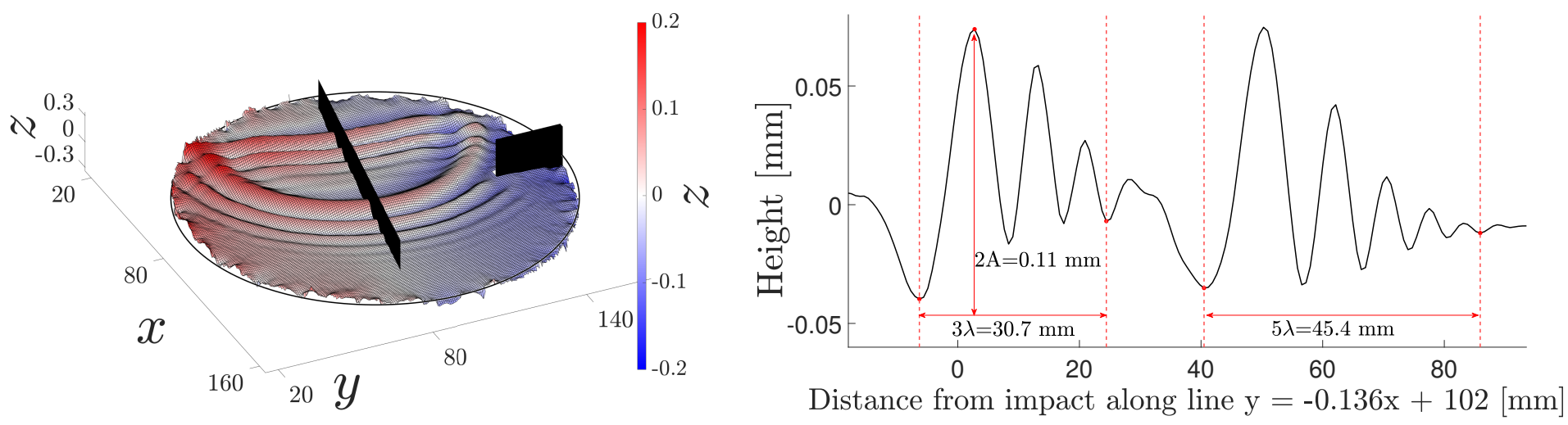

Fig. 1 (a) Fig. 4(b) from the main manuscript with a slice along y = -0.136x+102 mm through the impact location and wavefront. (b) Height profile of the interface along the slice, indicating the measured amplitude A and wavelength λ. All dimensions are in mm.

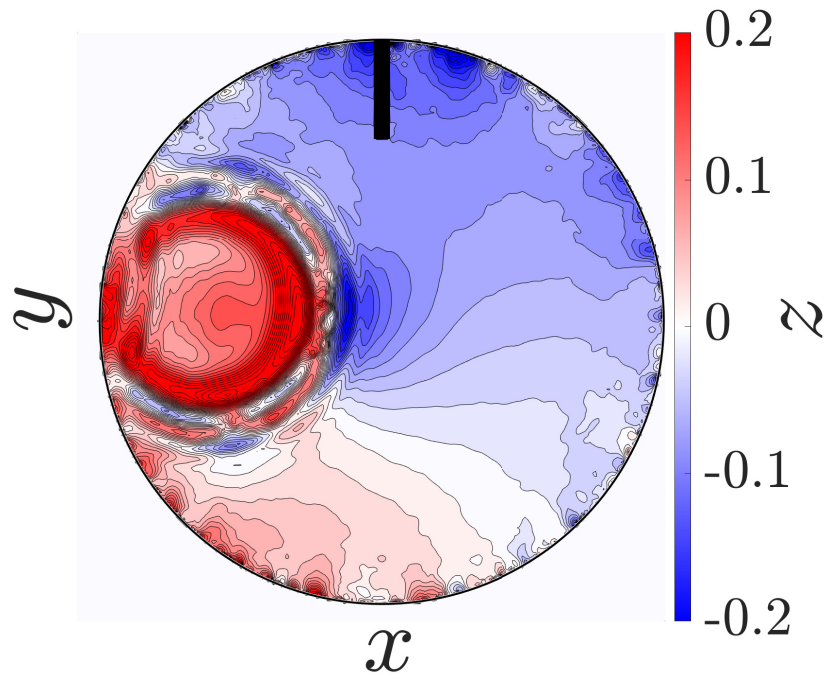

(a) $t = 0.13$ s

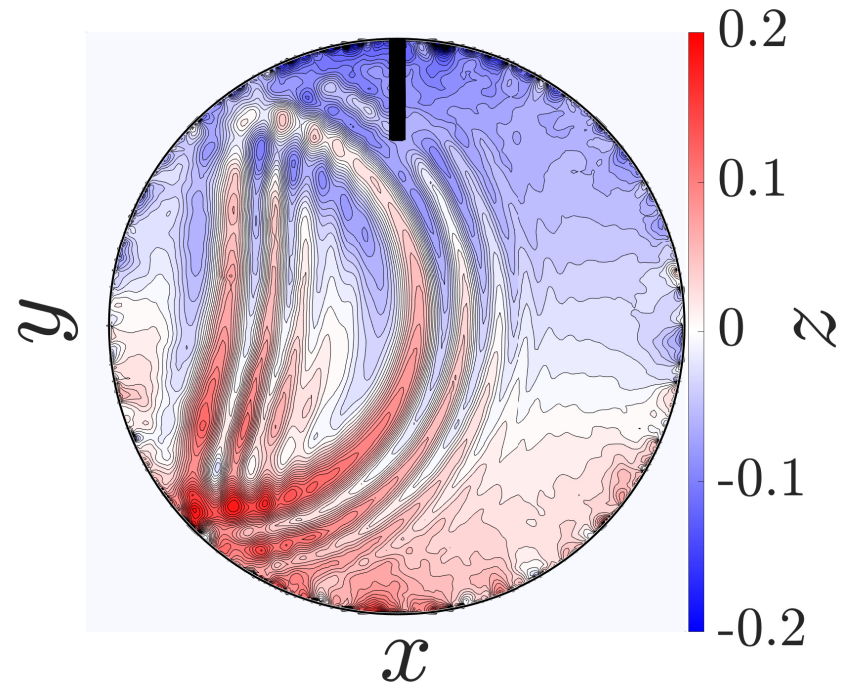

(b) $t = 0.27$ s

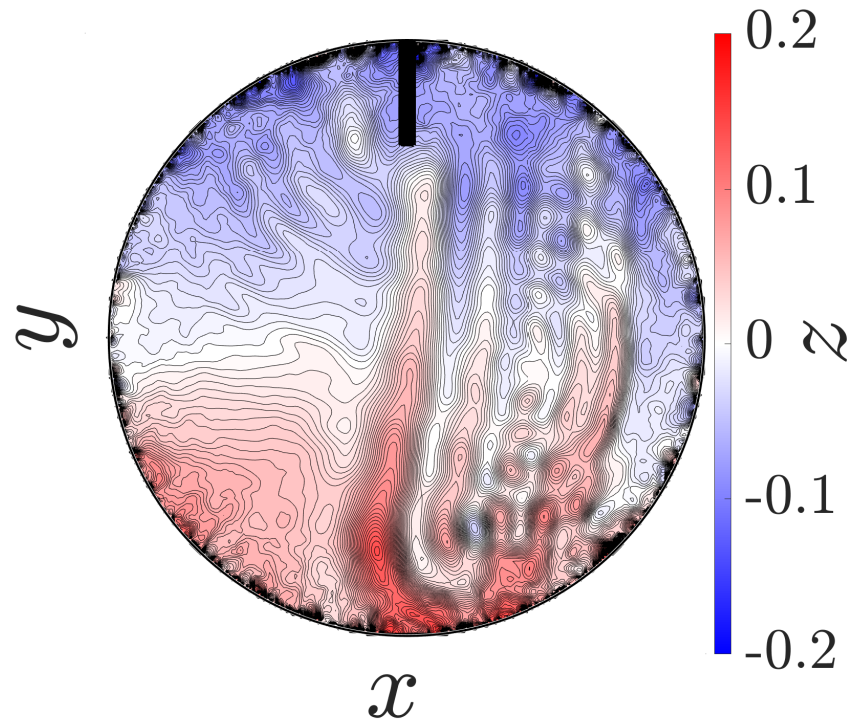

(c) $t = 0.57$ s

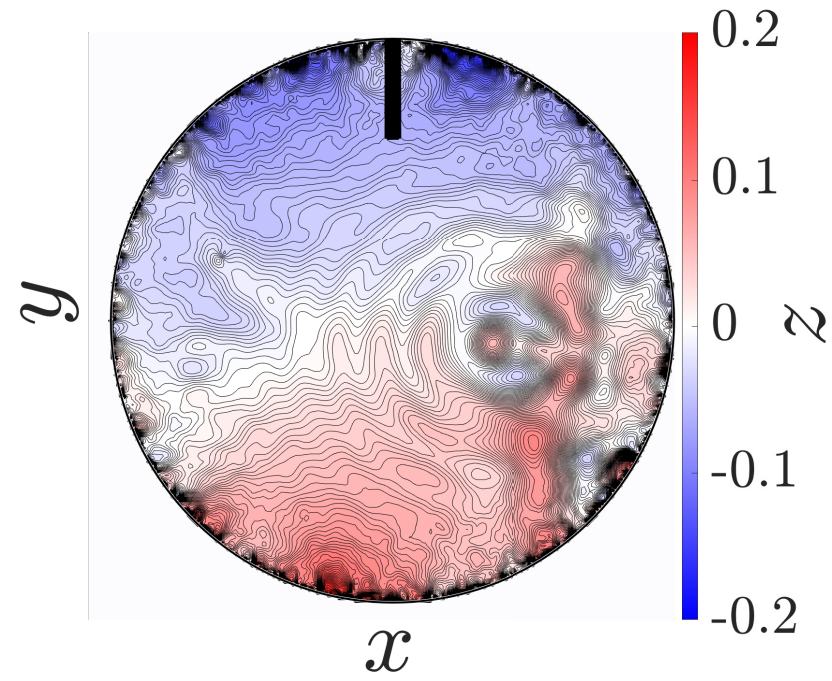

(d) $t = 0.87$ s

Fig. 2 Contour plot of Fig. 4 from the main manuscript. The color bar notes the measured water surface height in [mm] within the cylinder at $t = 0.13$, 0.27, 0.57, and 0.87 seconds after impact of the sphere with the water surface.

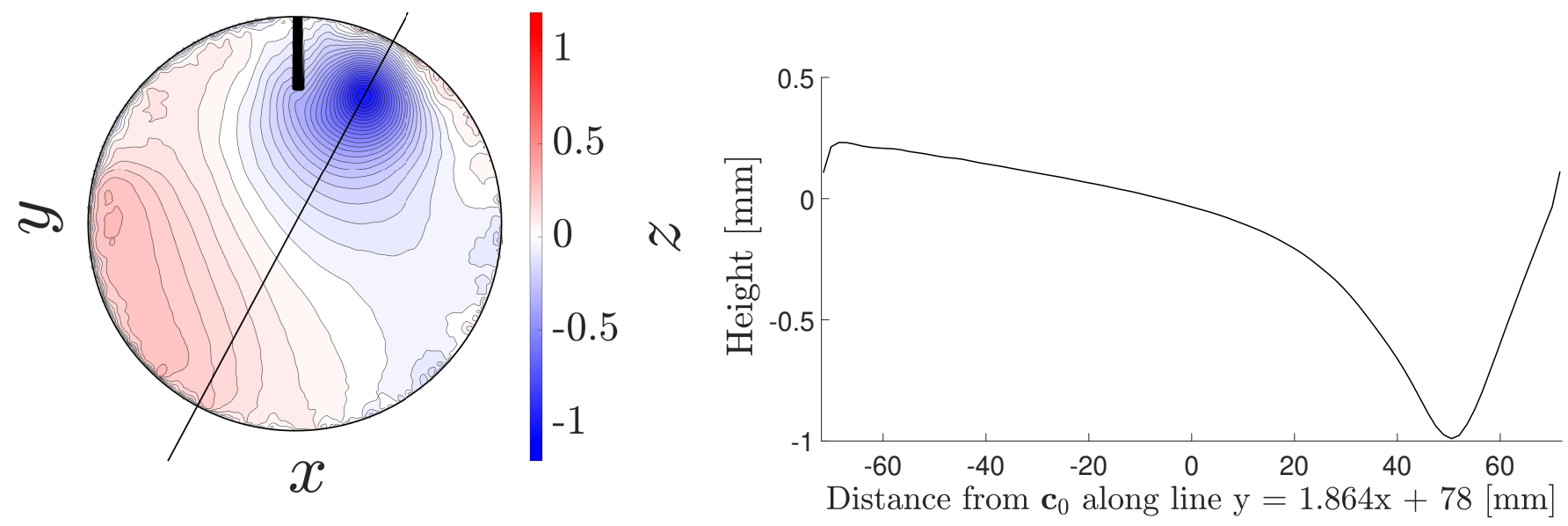

Fig. 3 (a) A contour plot of Fig. 5(a) with a slice along y = 1.864x+78 [mm] through $\mathbf{c}_0$ and the center of the vortex. (b) The height of the interface along the slice. All dimensions are in mm.
